# Nurses’ and midwives’ perspectives on participation in national policy development, review and reforms in Ghana: a qualitative study

**DOI:** 10.1186/s12912-021-00545-y

**Published:** 2021-01-22

**Authors:** Angela Kwartemaa Acheampong, Lillian Akorfa Ohene, Isabella Naana Akyaa Asante, Josephine Kyei, Gladys Dzansi, Charles Ampong Adjei, Samuel Adjorlolo, Francis Boateng, Philomena Woolley, Felix Nyante, Lydia Aziato

**Affiliations:** 1 School of Nursing, Wisconsin International University College-Ghana, P.O Box LG, Accra, Ghana; 2 School of Nursing and Midwifery, University of Ghana, Legon P. O Box LG 43, Accra, Ghana; 3 Isabella HealthCare Services, Accra, Ghana P. O. Box 10988,; 4 CSIR-Institute of Industrial Research, P.O. Box LG 576, Accra, Ghana; 5 Nursing and Midwifery Council of Ghana, Box MB 44 Ministries, Accra, Ghana

**Keywords:** Ghana, Health policy, Policy development, Policy review, Nurses, Midwives’, Africa

## Abstract

**Background:**

The World Health Organization has admonished member countries to strive towards achieving universal health coverage (UHC) through actionable health policies and strategies. Nurses and midwives have instrumental roles in achieving UHC via health policy development and implementation. However, there is a paucity of empirical data on nurses and midwives’ participation in policy development in Ghana. The current study explored nurses and midwives’ participation in policy development, reviews and reforms in Ghana.

**Methods:**

A qualitative descriptive exploratory design was adopted for this study. One-on-one individual interviews were conducted after 30 participants were purposefully selected. Data was audiotaped with permission, transcribed and analyzed inductively using the content analysis procedures.

**Results:**

Two main themes emerged from the data: participation in policy development and perspectives on policy reviews and reforms. The findings showed that during health policy development and reviews, nurses in Ghana were overlooked and unacknowledged. Policy reforms regarding bridging the pre-service preparation gap, staff development and motivation mechanisms and influence on admission into nursing schools were raised.

**Conclusion:**

The authors concluded that nurses and midwives are crucial members of the healthcare systems and their inputs in policy development and reviews would improve health delivery in Ghana.

**Supplementary Information:**

The online version contains supplementary material available at 10.1186/s12912-021-00545-y.

## Background

The World Health Organization has the mandate to ensure universal health coverage [1]. This goal can only be achieved if the contribution of nurses to healthcare systems is acknowledged and their suggestions and feedback on policies properly put into action. Nurses form the majority of human resource within every healthcare system globally; and this does not only put nurses at the frontline of health care delivery, but it also places them in a position where they have prolonged contact with the sick. Therefore, health care policies may directly or indirectly have an impact on nurses and their clients. Most patients, especially in some parts of Ghana have little to no access to physicians [2]. Consequently, they spend most of their time at the health facilities with nurses more than with any other health personnel. The consistent presence of nurses even across the hard to reach facilities allows them to have firsthand information on the challenges faced by clients within health facilities, making their participation in policy development and review crucial. In some high income nations, nurses can directly influence the development of policies [3], unfortunately, this scarcely occurs in the context of low and middle income nations [4].

There are facilitators and barriers to nurses’ participation in policy review and development. These include the image of nursing as well as structures and processes within health care systems [5]. Evidence shows that various power dynamics within the health sector hinder the participation of nurses in policy development [6]. Patriarchy is a power dynamic which operates with the principle of male dominance which relegates women directly or indirectly into the background [7–13]. In the African patriarchal context where women are mostly disadvantaged [14–17], the gender role of women in society may have an impact on the way nurses, majority of whom are women, are involved in policy development. Most often, nurses are not considered when policy committees are constituted [18]. This may be attributed to the fact that nursing is a female-dominated profession and policy development is considered as a leadership role. Meanwhile, women are typically not considered as leaders in patriarchal societies [19]. Anecdotally in the Ghanaian context, nurse leaders can only exert their influence on nurses and not the entire health system since health service leadership is mostly occupied by medical staff. Therefore, their involvement in policy development and review is only limited to policies concerning nurses. This limits their involvement in national policies that concern the entire health system.

Some experts in nursing have advocated that the way forward is to train, educate, and mentor nurses in the area of policy development and review [20, 21]. Irrespective of these efforts, individual nurses must demonstrate some leadership attributes such as effective communication, empowerment and good interpersonal skills to merit inclusion in policy development and review activities [22]. Empowerment of nurses and nurse leaders makes them proactive and advocates in the health sector which are great attributes for contribution to policy development and review [23–25]. Nurses can also influence policy development and review effectively if they participate in politics at their local communities [26, 27]. Nurses should be empowered to be proactive in the issues of policy development [25]. The empowerment process should commence by understanding existing practices and issues pertaining to health policy development and reviews. This way, limited resources can be directed and utilized to achieve the maximum. More importantly, although previous studies have offered some useful insights with respect to nurses/midwives’ participation in policy development, it should be noted that their usefulness in reengineering Ghanaian health system may be limited. As noted by [28] Adjorlolo et al., the structural and functional differences in the organization and delivery of healthcare services across countries calls for context specific understanding of global issues. Doing so would not only contribute to the needed scholarship on health policy development but enhance and advance cross-cultural discourses. Consequently, this study explored the contribution and/or participation of Ghanaian nurses and midwives in health policy development, reviews and reforms in Ghana.

## Methods

### Aim, design and setting

This study aimed at exploring nurses’ and midwives’ participation in policy review and development. The study employed a qualitative descriptive exploratory design. This design suited the study because it afforded participants the opportunity to fully describe their perceptions and allowed the researchers to probe the subject matter under study. At the time of data collection, Ghana had ten administrative regions. The study was conducted in six of those regions; Volta, Western, Greater Accra, Ashanti, Upper East, and the Northern Regions of Ghana which fall under the three main zones of the country. The zones being the Northern, Middle and Southern. This method allowed nurses and midwives across the country to voice their opinions on the subject matter. Participants from different employment settings for nurses and midwives such as hospitals, educational institutions, health directorate offices and the Nursing and Midwifery Council of Ghana were selected for the study. This offered the research teams the opportunity to understand nurses’ and midwives’ participation in policy development and review across diverse settings.

### Sampling and data collection procedures

Thirty (30) nurses and midwives were recruited into the study since data saturation was reached on the thirtieth participant. Fifteen (15) of the participants were general nurses and fifteen (15) were midwives. The participants were purposively selected based on the leadership roles they played in their places of work. The criteria for inclusion into the study were; a nurse leader in a key position who voluntarily accepts to be part of the study. Prospective participants were given information sheets that contained information about the study. They were then contacted after several days to enquire if they would like to participate in the study. Those who voluntarily opted to be part were then given consent forms to fill. Participants spoke English during face-to-face interviews as English is the official language in Ghana. A semi-structured interview guide (Refer to Additional file 1) which was developed specifically for this study based on the study objectives was used to collect data. Open-ended questions were to allow free expression. Some questions asked included: *what are your views on nurses’ and midwives’ participation in national policy development and review? Which national health policies do you think need reforms and why?* The interview guide was pilot tested among three (3) nurses in a separate health facility for ambiguous questions to be clarified. Times and venues for the interviews were at the convenience of the participants. Interviews were conducted in such a way that privacy was ensured with only the interviewer and interviewee at secluded rooms. Interviews were collected at participants’ workplaces and each interview lasted between thirty (30) minutes and an hour. Verbatim transcriptions were done after interviews were audiotaped. Data collection stopped only after saturation was reached where no new information was obtained.

### Data analysis

Concurrent data collection and analysis ensured that themes and sub-themes that emerged from previous interviews were probed in subsequent ones. Content Analysis was the choice of analysis and data analysis was done following the steps described by Padgett [29]. Initially, data cleaning was done by removing all identifiable names and places from transcripts. Transcripts were read and re-read several times to have a deep understanding of participants’ perspectives before words and phrases were attached to sentences (coding). The codes were such that, they captured the meanings of participants’ perspectives. Similar codes were aggregated at this point to form sub-themes. Major themes finally emerged after sub-themes were put together. The team of researchers met at different points of data analysis to discuss emerging themes and sub-themes. At such meetings, the researchers assessed the emerging themes and sub-themes and made inputs to refine them. This allowed everyone in the team to ensure that participants’ perspectives were maintained.

### Trustworthiness of the study

Verbatim transcription of data ensured that participants’ perspectives were kept intact. Some of these verbatim quotes have been used to support the findings and this has given a voice to the nurses and midwives. Since data collection and analysis were done concomitantly, it allowed the researchers to probe emerging themes and sub-themes in subsequent interviews. For clarification, some participants were contacted for member checking where necessary. The same interview guide was used to collect data from all participants from the different parts of the country. Field notes which were taken during and after data collection were used as backups during data collection and this ensured that information collected was verified. Field notes were compared with transcripts to ensure that the data was not distorted.

### Ethical considerations

Ethical clearance was obtained from the Ghana Health Service Ethics Review Committee (GHS-ERC 011/05/19). To ensure voluntary participation in the study, participants were asked to give their consent before partaking in the study. During data collection, the authors ensured that they were non-judgmental and anti-prejudice. Data were audiotaped with permission from participants and they were assured that they could withdraw from the study at any time without any repercussions. Participants’ identities were kept intact by representing them with codes devoid of any form of identification thereby ensuring anonymity.

## Results

### Demographic data

The objective of this study was to explore nurses’ and midwives’ participation in policy review and development. Thirty (30) nurse leaders were interviewed. Eleven (11) out of the thirty (30) were males and nineteen (19) were females. Participants’ ages ranged from 31 to 58 years. Seven (7) of the participants had diploma as their highest qualifications, seventeen (17) had first degree as their highest qualifications and six of them had master’s degrees as their highest qualifications. All the participants occupied key leadership positions at their places of work. Their positions ranged from hospital unit heads to heads of health institutions.

### Themes

Two main themes and six sub-themes emerged from the data (Refer to Table 1). The main themes included; Participation in national health policy development and perspectives on policies that need reforms.

**Table 1:** Emerging Themes, Subthemes and Codes

Theme	Subthemes	Codes
Participation in national health policy development	Nurses and midwives being overlooked and unacknowledged	All doctors Involved Seek inputs Not recognized Not mentioned Seek opinions Not named
	Etic and emic of nurses	Not knowledgeable Not intelligent Least knowledgeable No impact Not recognized No good material Poor image Kowtowing Historical image Female dominated
Perspectives on policies that need reforms	Pre-service preparation gap	Few clinicians Poor foundation Strong collaboration Eager to school Inexperience Being skillful Correction Interactive teaching Inexperienced teachers Practical exposure
	Influence on admission into nursing schools	Selection into schools Student recruitment Penetration into profession Job opportunity Political manipulation Politics and nursing Higher authority
	Staff development and motivation mechanisms	Leaders not nurses Unfair treatments Unrecognized certificates Low salary No study leave

### Participation in national health policy development

This theme describes the participation of nurses and midwives in policy development at the national level. Two subthemes emerged from this; Nurses being overlooked and unacknowledged and Etic and emic of nurses.

### Nurses being overlooked and unacknowledged

According to most participants, nurses and midwives have the experience and skills and are involved in some national policy development activities in areas such as reproductive health, code of ethics, health bill, acute emergency care and policies on nursing and midwifery curriculum development. Even though they are involved in developing some policies, few of them are invited as compared to the medical doctors who form the majority of the policy development team and the few that are involved are mostly not acknowledged as authors of the policy document.

*“…about three years ago I was called to join the team where we would have the reproductive review policies done… almost all of them were doctors and that was what saddens my heart… After the document came out, I was not even acknowledged”* (P11-Midwife)

*“I mean, I remember the health bill, I was part of it but I was not really recognized, but, most of the other team members were medical doctors”* (P4-Nurse)

A participant narrated that before a policy is developed or reviewed, the grass-root which majority are nurses and midwives must be consulted because they are the implementers but that is not the case in Ghana.

*“I think that, since nurses and midwives are the implementers of most health policies, we should be given the chance to have a major voice in the development of health policies at the national level”* (P15-Nurse)

It was emphasized that even though some nurses and midwives are involved in policy development, they are mostly not recognized. The credit is mostly given to medical doctors and that makes them feel that they are not part of the team.

“…*they will mostly seek your inputs during the development process but at the end of the day …. you won’t see or hear your name once the policy is developed”* (P21-Midwife)

One participant stated that, the office that was given to nurses for policy development at the ministry was not even recognized officially.

*“At the Ministry of Health in Ghana, though we used to have an office for nurses that under normal circumstance is to be involved in policy development and other issues for nurses and midwives specifically, it is even not legitimately recognized”* (P7-Nurse)

Some also disagreed with this assertion and believed that recognition would be given to a nurse during policy development depending on the perceived value of his or her contribution which draws attention to the need for nurses and midwives to strive to make their voices heard at the policy development table.

*“I disagree to a large extent when nurses perceive that, they are not recognized during policy development. It depends on… the value you place on yourself. For example, you just saw a letter being brought to me to go and represent the health ministry. Why will the minister ask a nurse to go when there are doctors?”*(P28-Nurse)

### Etic and emic of nurses

Some of the participants indicated that nurses and midwives were not involved in national policy development and review and cited various reasons for this non-participation such as inadequate knowledge in policy formulation in the substantive areas of policy being developed, being inferior to other health professionals and non-participation in politics.

*“I think that we nurses are perceived not to be knowledgeable… So the public assume that “oh as for nurses... they are not intelligent" they even respect teachers more than us when it comes to the level of policy development* (P19-Midwife)

*“Yeah, we nurses are not knowledgeable. That is the perception. Not publicly but … comparing us with other health care workers, the nurses are considered as least knowledgeable”* (P24-Midwife)

*“Nurses shy away from policy development because one, they don’t have the knowledge and two, they don’t think it is important for them to participate in the policy process”* (P15-Nurse)

Some participants attributed the perceived lack of knowledge among nurses and midwives to the low educational level. They therefore called on nurses and midwives to strive to pursue higher education.

*“I think it has to do with the educational level of the nurse, some nurses have not been… trained to be nurses…and even people go in for a degree and they still lack knowledge in policy development so they should pursue further studies”* (P8-Midwife)

Some participants said even if nurses are called to participate in policy development or review, they would not make any impact because they lack knowledge in the substantive areas that the policy focuses on.

*“But in terms of policy, we nurses don’t make any impact. … even when the principals of nursing schools are invited into policy review meetings, they all listen quietly to the minister of health, whatever he says is final and they don’t make any impact. They can’t question anything because they don’t know”* (P17-Nurse)

Some participants were of the view that there were nurses who could help develop health policies, but the policymakers did not invite them. Most top positions in the Ministry of Health are held by medical doctors who represent health workers at the national policy development table.

*“I think it’s because the authorities that see to this policy development have not yet recognized nursing and midwifery. …if you get to clinical practice, the highest person in nursing at that unit, works within the clinical care and that unit is headed by a medical doctor. Go to public health department and the highest rank in nursing in public health is under a medical doctor, So, if there is any policy development it is the top person there who would be part”* (P25-Nurse)

The history of nursing is also identified to affect the way nurses are treated in Ghana as people still perceive nurses not to be on top of issues even though nurses have acquired higher degrees including terminal degrees. With nurses and midwives being professors and doctors, some participants believed that, policy developers must involve nurses and midwives in developing and reviewing national health policies.

*“Basically, it has historical antecedents. It is about the perception people have that we nurses don't have …good… material that will be able to make input in national policy development or decision making. Because of this perception and ignorance on the part of the citizenry and even policy formulators, they tend not to rely on nurses…for their input”* (P14-Nurse)

*“…it saddens my heart that gone are the days when they thought nurses and midwives should sit behind the benches …It is because of the old historical image. It saddens my heart” (*P11-Midwife)

According to a participant, the way nurses and midwives are trained also influences their confidence and assertiveness for fear of victimization and deprivation of opportunities.

“*Nurses end up kowtowing and then keeping to their shells because of the fear that when they speak out, they will be deprived of certain opportunities and so they will not speak. And so the doctors will continue to bully us”* (P15-Nurse)

The gender disproportions in nursing where females form the majority were identified as a key factor that prevented nurses from holding national leadership positions in the health sector in Ghana. Almost all the participants asserted that policy development is a leadership role and nursing being a female majority is perceived to play subservient roles only.

*“…it saddens my heart that gone are the days when they thought nurses and midwives should sit behind the benches …It is because of the old historical image of females and nursing being a female-dominated profession is seen as a subservient role. So how will they take us seriously and involve us in policy development? Because policy development is seen as a leadership activity”* (P11-Midwife)

*“Throughout history and now, nursing has always been female-dominated and females are not respected in our part of the world. Therefore, no one thinks that anything good can come from females so why would they involve us in national policy development?”* (P25-Nurse)

### Perspectives on policies that need reforms

This theme generated sub-themes such as: Pre-service preparation gap, influence on admission into nursing schools as well as staff development and motivation mechanisms.

### Pre-service preparation gap

Participants perceived that, there was a theory-practice gap in the pre-service preparation of student nurses before they entered into the profession. Both clinicians and educators lamented about the phenomenon. While the nurse educators complained that clinicians did not teach students during clinical practice, clinicians also narrated that, students were also taught by inexperienced tutors/lecturers who were unable to have impact on the students as expected.

*“…there are few clinicians who are willing to help or supervise students, so we all contribute to some of these things and when they graduate it becomes a bit difficult because the foundation would not be that good”* (P22-Midwife)

*“Most student nurses are taught by inexperienced tutors/lecturers who end up making no impact in their training”* (P25-Nurse)

Some participants called for policies that would ensure strong collaboration between nurse clinicians and educators to improve clinical practice.

“*Yes, I think there should be policies that would ensure strong collaboration between the clinical side and the educational institutions to help improve the competency so that we don’t train incompetent nurses”* (P17-Nurse)

It was reiterated that the theory-practice gap problem could be solved if nurses were obliged by certain policies to work in the clinical area for some years before pursuing higher education.

*“…as soon as the person finishes nursing school, that same year he/she starts schooling again. He/she is not eager to work. …almost all the nurses are on night shift. Some have finished their RGN, and already have masters’ degree... So they are not focused on picking the skills and growing with it”* (P23-Midwife)

All the participants strongly suggested that, the theory-practice gap should be bridged.

“*…the care at the clinical area is nothing to write home about and so that in itself paints a particular picture. We claim we are academically endowed but when it comes to the clinical work we are lacking; so, this must be corrected with all seriousness*” (P24-Midwife)

A participant compared the dual role medical doctors play as lecturers and clinicians which she recommended should be emulated by nurses and midwives.

“W*hen you take medical school, you would have the same consultants or clinicians who are teaching as adjunct lecturers or full time at the university… that is more of interactive teaching and learning, but our module of teaching in nursing education is not in that format and so that is where the gap which needs to be bridged…”* (P3-Nurse)

Others also complained about the number of years nurses spend on the ward before joining academia. Some complained that most of the nurses and midwives teaching at the various training institutions are not practically inclined to be able to teach the students.

*“There are a lot of people teaching in nursing schools; those teachers themselves are not skilled practically, they did not have any practical exposure and so it is difficult for them to demonstrate the practical component of the courses…*” (P29-Midwife)

*“I think from the training if we have experienced tutors teaching them and adding these morals that we were taught, I believe it will help a lot”* (P9- Nurse)

### Influence on admission into nursing schools

This sub-theme describes how people in governance and other higher offices influence the admission of people into nursing training institutions. Participants reported that most of the people who are admitted into the schools of nursing are not interested in nursing and midwifery but are pushed into it by their members of parliament as a way of getting them jobs. They called for the need for a stringent admission policy.

*“When it* comes *to the selection of people into these training colleges, they are manned by Ministry of Health, the protocol bit sometimes is killing and so we have people who are enrolled in these schools who may necessarily not have the passion and are sort of compelled….” (P15-Nurse*)

Some believe that politics has taken over nursing education. Politicians are said to have used nursing institutions as a job creation avenue for their constituents. Politicians, according to some participants have capitalized on nurses’ weakness to infiltrate the profession. This limits the authority of nursing and midwifery leaders to make policy reviews or reforms.

*“…politicians* began *penetrating into the profession and it also became an avenue for job creation…Politicians felt that when they push people into nursing, then it's like they have created employment or a job opportunity for their constituents”* (P14-Nurse)

*“…when I saw that the registrar of N&MC withdrew that letter which was meant to stop the training of* auxiliary *nurses, I was like; is this man having his own power to work or is he politically being manipulated? Our current situation is such that politics has taken the center of nursing education”* (P3-Nurse)

The students who are admitted through protocol are said to disobey school rules. There are times higher powers step in when these students misbehave and prevent lecturers from disciplining them. Thus, if there were clear policies and there was freedom to implement them, these issues will not arise.

*“… If a student* does *something wrong and the tutor tries to punish that student, because the student is connected to a higher authority in the school, that student may report to the higher authority. So, we need policies on all these and the freedom to implement them”* (P2-Midwife)

The number of students admitted into the training institutions is said to be too much, and for which reason, they think it negatively affects the quality of teaching and learning. According to participants, those who are admitted through protocol are not screened properly because their interviews are just formalities.

*“Some schools admit 2000 students, what type of quality teaching is ongoing? So eventually, they graduate and cannot perform*” (P17-Nurse)

### Staff development and motivation mechanisms

This sub-theme expounds nurses’ and midwives’ concern about their working conditions, promotions and how to climb to leadership positions to get their voices heard in policy development and avoid being disrespected by other members of the healthcare team.

Clinical nurses and midwives said although they work tediously under stressful conditions, medical doctors are given priority treatment and when they try to question such unfair treatment, they are victimized by hospital management with transfers. To them, such treatment could be abated if more nurses are found at the policy table.

*“There was a midwife who renovated the hospital’s residence on her own. When the room was completed, she moved in for about just three weeks or a month and this same midwife now had a message that, they now have a doctor who had accepted to be resident so she should move out. This would not have happened if those at the hem of policy development were nurses”* (P29-Midwife)

*“Doctors are given accommodation; they are given allowances and everything but nurses are left out due to our inability to be part of policy development”* (P5- Midwife)

*“…where I live is* quite *far from this place, but then there is a hospital bus that goes around to pick just* doctors *and then the management, yes so I have to pick three cars before getting to this place every day, and this is because few nurses are part of policy development” (*P5-Midwife)

Nurses who further their education on their own are being demoralized because after they struggle to use their annual leaves and weekends to school, their certificates are not recognized by the Ghana Health Service for promotion.

“…*look at the universities all over somebody would even go with leave, they present certificate for promotion, they would tell you this one we don’t recognize it because we did not give you study leave, my sister can you imagine this how painful it is and now the young ones are crying within them*” (P11-Midwife)

*“…you see that our salary is quite low, yes our salary is quite low and all these can change if nurses are promoted to participate in policy development”* (P26-Nurse)

## Discussion

The key findings from this study revealed that only few nurses participated in policy development and review, and these contributions were often undocumented. Evidence shows that nurses are not recognized most of the time due to general invisibility and the lack of discourse in the public space [30]. Therefore, nurses and midwives must show gentle aggressions when participating in policies that directly and indirectly affect their profession and clients. It is presumed that their visibility in the media will enable the public to bear witness to their advocacy activities.

Nurses’ non-participation in policy development as reported in this study resonates with findings from other studies [31, 32]. Other scholars have attributed these phenomenon to a lack of autonomy in the nursing profession [33–37]. Furthermore, the evidence of critical thinking and good judgment skills as facilitating factors of nurses’ participation in policy development have been reported [5]. This has been echoed in other studies where members of the public considered the nature of nursing education as basic, task oriented, and therefore fallen short of analytical skills [38, 39]. It is therefore not surprising that among student nurses, some have reported that they chose to enter the nursing/midwifery profession because of its low admission requirements relative to that of medical students [40]. Perhaps, raising the entry requirements levels into the nursing profession would erase the existing poor image. Some participants felt that the female majority in the nursing profession is also a factor that excludes them from being involved in policy development. This may be due to the patriarchal nature of the Ghanaian society where most decision-making positions are held by males. Efforts should be made to change this narrative by training nurses to be advocates or campaigners in health delivery systems. Meanwhile, nurses can be good advocates change if they are empowered at their work settings [41–44]. Various change theories have been documented to effect change in behaviour [45–48] and therefore should be applied to cause behaviour change.

Pre-service preparation gap is one of the key areas nurses desire policy change. It was evident from the findings that the transition between classrooms and the clinical field is among the challenges of nursing practice in Ghana. Both nurse clinicians and academics blamed each other for the poor transition of the novice nurse to the nurse with expertise. The presence of a pre-service preparation gap in nursing and midwifery has been documented vastly in literature [49–57]. Suggestions in earlier research reports on the way to bridge such gaps included effective simulation in well-equipped skills laboratories [50, 51, 54, 57], nurse leaders combining personal and organizational empowerment strategies to empower novice nurses [58], effective communication between academics and clinicians [52, 53] as well as recognizing the critical role of the clinical instructor in the training of nurses [49, 53]. These suggest that academics in nursing education in Ghana must consider working concurrently in the clinical areas, where possible. Both clinicians and academics should apply learning theories that would affect positive learning attitudinal change in learners [59]. This would ensure effective collaboration between clinicians and academics and consequently lead to the bridging of the theory-practice gap.

Nurses and midwives who participated in this study wished that policies that would prevent politicians from influencing the nursing profession could be formulated. It is believed that this would prevent the recruitment of unqualified candidates into the profession. This challenge is achievable if nurses can exhibit negotiable skills, heightened by personal attributes such as persistence, fortitude, willpower, and resilience. Unfortunately, [60, 61] have reported the lack of negotiable skills among nurses which generally laid nurses back in political discourse. This could be due to the approach of training given to nurses in the Ghanaian context which predisposes them to timidity and unassertiveness. The nursing and midwifery curricula in Ghana must include aspects that teach skills like negotiation and assertiveness.

Finally, the participants in this study re-iterated a need for equitable policies on empowerment, promotion, and motivation. The inequalities that exist in the promotion and motivation of nurses, as well as midwives could be due to the hostilities among senior nurses which lead to power struggles within the nursing fraternity [61]. Consequently, nurses and midwives should be reminded at all times that, they form the majority of the health workforce. Being the majority gives them a huge advantage in terms of the formation of a formidable force that can advocate for better conditions of service if only they remain united.

## Conclusion

It was evident that nurses appreciate policies and regulations as guiding elements for the smooth operations of any healthcare system. In this study, nurses and Midwives hinted at their limited participation in policy development in the health sector in Ghana. In a few instances where nurses attested to their participation in policy development and review, the evidence of nursing contribution mostly was unrecognized. This study further advocated for policies in areas such as; pre-service preparation gap, avoiding influence in nursing education and duly motivating nurses/midwives. Based on these findings, the authors recommend a bachelor degree as the entry point of nursing and midwifery profession in Ghana to conform with the international standards and practice. This will contribute to achieving the desired positive image of the profession in the Ghanaian context. Also, a roadmap to policy reforms regarding the elimination of excessive political influence in training nurses, increased inter-professional education and thoughtful inclusion of nurse/midwives in policy related discussions is required. Future research should focus on how nurses and midwives participate in policy reform processes in Ghana.

## Supplementary Information

**Additional file 1.** Qualitative interview guide.

## Data Availability

The datasets analyzed during the current study are available from the corresponding author on reasonable request.

## Abbreviations

UHC: Universal Health Coverage
GHS-ERC: Ghana Health Service Ethical Review Committee

## References

[1] W. H. Organization, A vision for primary health care in the 21st century: towards universal health coverage and the Sustainable Development Goals. Geneva: World Health Organization and the United Nations Children’s Fund (UNICEF); 2018 (WHO/HIS/SDS/2018.15). Licence: CC BY-NC-SA 3.0 IGO.

[2] Bonenberger M, Aikins M, Akweongo P, Wyss K (2014). The effects of health worker motivation and job satisfaction on turnover intention in Ghana: a cross-sectional study. Hum Resour Health.

[3] Gregg J. Influencing policy to reduce motor vehicle crashes in a rural community: a multiple streams approach, PhD Thesis, Carlow University; 2017.

[4] AbuAlRub RF, Abdulnabi A (2020). Involvement in health policy and political efficacy among hospital nurses in Jordan: a descriptive survey. J Nurs Manag..

[5] Shariff N (2014). Factors that act as facilitators and barriers to nurse leaders’ participation in health policy development. BMC Nurs.

[6] Asuquo E, Etowa JB, Gifford WA, Holmes D (2016). Nurses’ involvement in HIV policy formulation in Nigerian health care system. J AIDS Clin Res.

[7] Akgul F. Understanding Patriarchy. In: Patriarchal Theory Reconsidered. Palgrave Macmillan, Cham. 2017. 10.1007/978-3-319-49766-2_3.

[8] Bui HT, Kuan A, Chu TT (2018). Female entrepreneurship in patriarchal society: motivation and challenges. J Small Business Entrepreneurship.

[9] Chigbu UE (2019). Masculinity, men and patriarchal issues aside: how do women’s actions impede women’s access to land? Matters arising from a peri-rural community in Nigeria. Land Use Policy.

[10] Kahalon R, Bareket O, Vial AC, Sassenhagen N, Becker JC, Shnabel N (2019). The Madonna-whore dichotomy is associated with patriarchy endorsement: evidence from Israel, the United States, and Germany. Psychol Women Q.

[11] Mustafa M, Batool A, Fatima B, Nawaz F, Toyama K, Raza AA (2020). Patriarchy, Maternal Health and Spiritual Healing: Designing Maternal Health Interventions in Pakistan. Proceedings of the 2020 CHI Conference on Human Factors in Computing Systems: 2020.

[12] Salem R, Yount KM (2019). Structural accommodations of patriarchy: women and workplace gender segregation in Qatar. Gender Work Organization.

[13] Zhao EY, Wry T (2016). Not all inequality is equal: deconstructing the societal logic of patriarchy to understand microfinance lending to women. Acad Manag J.

[14] Alozie NO, Akpan-Obong P (2017). The digital gender divide: confronting obstacles to women’s development in Africa. Dev Policy Rev.

[15] Dichabe D. Eradicating the patriarchal state: promoting women’s socio-economic rights and achieving gender equity in the economic participation of women in South Africa (1994–2017), PhD Thesis, University of the Free State, 2017.

[16] Folbre N. Patriarchal social formations in Zimbabwe’, In: Stichter, Sharon B. and Parpart, Jane L. (eds.). Patriarchy and Class: African Women in the Home and the Workplace. Boulder: Westview Press; 1988. p. 61–80.

[17] Hoover A. “War within the war”: overcoming a legacy of patriarchal norms and violence in the Central African Republic and the Democratic Republic of Congo. Global Majority E-J. 2019;57.

[18] Juma PA, Edwards N, Spitzer D (2014). Kenyan nurses involvement in national policy development processes. Nurs Res Pract.

[19] Mwale C, Dodo O (2017). Sociocultural beliefs and women leadership in Sanyati District. J Soc Change.

[20] Kunaviktikul W (2014). Moving towards the greater involvement of nurses in policy development. Int Nurs Rev.

[21] Turale S, Kunaviktikul W (2019). The contribution of nurses to health policy and advocacy requires leaders to provide training and mentorship. Int Nurs Rev.

[22] Shariff NJ (2015). A Delphi survey of leadership attributes necessary for national nurse leaders’ participation in health policy development: an east African perspective. BMC Nurs.

[23] García-Sierra R, Fernández-Castro J (2018). Relationships between leadership, structural empowerment, and engagement in nurses. J Adv Nurs.

[24] Read EA, Laschinger HK (2015). The influence of authentic leadership and empowerment on nurses’ relational social capital, mental health and job satisfaction over the first year of practice. J Adv Nurs.

[25] Shariff NJ (2015). Empowerment model for nurse leaders’ participation in health policy development: an east African perspective. BMC Nurs.

[26] Wang H-H (2014). “ Scholar officials”: thoughts on the involvement of professional nurses in the political process. Hu Li Za Zhi.

[27] Woodward B, Smart D, Benavides-Vaello S (2016). Modifiable factors that support political participation by nurses. J Prof Nurs.

[28] Adjorlolo S, Aziato L, Akorli VV (2019). Promoting maternal mental health in Ghana: an examination of the involvement and professional development needs of nurses and midwives. Nurse Educ Pract.

[29] Padgett DK (2011). Qualitative and mixed methods in public health: SAGE publications.

[30] Hoeve Y, Jansen G, Roodbol P (2014). The nursing profession: public image, self-concept and professional identity. A discussion paper. J Adv Nurs.

[31] Catallo C, Spalding K, Haghiri-Vijeh R (2014). Nursing professional organizations: what are they doing to engage nurses in health policy?. SAGE Open.

[32] Cheraghi MA, Ghiyasvandian S, Aarabi A (2015). Iranian nurses’ status in policymaking for nursing in health system: a qualitative content analysis. Open Nurs J.

[33] AllahBakhshian M, Alimohammadi N, Taleghani F, Nik AY, Abbasi S, Gholizadeh L (2017). Barriers to intensive care unit nurses’ autonomy in Iran: a qualitative study. Nurs Outlook.

[34] Amini K, Negarandeh R, Ramezani-Badr F, Moosaeifard M, Fallah R (2015). Nurses’ autonomy level in teaching hospitals and its relationship with the underlying factors. Int J Nurs Pract.

[35] Atefi N, Abdullah K, Wong L, Mazlom R (2014). Factors influencing registered nurses perception of their overall job satisfaction: a qualitative study. Int Nurs Rev.

[36] Galbany-Estragués P, Comas-d’Argemir D (2017). Care, autonomy, and gender in nursing practice: A historical study of nurses’ experiences. J Nurs Res.

[37] van Oostveen CJ, Mathijssen E, Vermeulen H (2015). Nurse staffing issues are just the tip of the iceberg: a qualitative study about nurses’ perceptions of nurse staffing. Int J Nurs Stud.

[38] Glerean N, Hupli M, Talman K, Haavisto E (2017). Young peoples’ perceptions of the nursing profession: an integrative review. Nurse Educ Today.

[39] Koo M, Lin SC (2016). The image of nursing: a glimpse of the internet. Jpn J Nurs Sci.

[40] Marcinowicz L, Owlasiuk A, Slusarska B, Zarzycka D, Pawlikowska T (2016). Choice and perception of the nursing profession from the perspective of polish nursing students: a focus group study. BMC Med Educ.

[41] Choi SL, Goh CF, Adam MBH, Tan OK (2016). Transformational leadership, empowerment, and job satisfaction: the mediating role of employee empowerment. Hum Resour Health.

[42] Dahinten V, Lee S, MacPhee M (2016). Disentangling the relationships between staff nurses’ workplace empowerment and job satisfaction. J Nurs Manag.

[43] Li H, Shi Y, Li Y, Xing Z, Wang S, Ying J, Zhang M, Sun J (2018). Relationship between nurse psychological empowerment and job satisfaction: a systematic review and meta-analysis. J Adv Nurs.

[44] Van Bogaert P, Peremans L, de Wit M, Franck E, Timmermans O, Havens DS (2015). Nurse managers’ perceptions and experiences regarding staff nurse empowerment: a qualitative study. Front Psychol.

[45] Barley E, Lawson V (2016). Using health psychology to help patients: theories of behaviour change. Br J Nurs.

[46] Corace KM, Srigley JA, Hargadon DP, Yu D, MacDonald TK, Fabrigar LR, Garber GE (2016). Using behavior change frameworks to improve healthcare worker influenza vaccination rates: a systematic review. Vaccine.

[47] Kwasnicka D, Dombrowski SU, White M, Sniehotta F (2016). Theoretical explanations for maintenance of behaviour change: a systematic review of behaviour theories. Health Psychol Rev.

[48] Srigley J, Corace K, Hargadon D, Yu D, MacDonald T, Fabrigar L, Garber G (2015). Applying psychological frameworks of behaviour change to improve healthcare worker hand hygiene: a systematic review. J Hosp Infect.

[49] Akram AS, Mohamad A, Akram S (2018). The role of clinical instructor in bridging the gap between theory and practice in nursing education. Int J Caring Sci.

[50] Botma Y (2014). Nursing student’s perceptions on how immersive simulation promotes theory–practice integration. Int J Africa Nurs Sci.

[51] McGill R, Anderson J, Francis K (2014). An innovative approach to nursing education: bridging the theory practice gap using simulated learning. Australian Nurs Midwifery J.

[52] Saifan A, AbuRuz ME, Masa’deh R. Theory practice gaps in nursing education: a qualitative perspective. J Soc Sci/Sosyal Bilimler Dergisi. 2015:11(1).

[53] Saifan AR, Safieh HA, Milbes R, Shibly R (2015). Suggestions to close the gap in nursing education: nursing students’ perspectives. Int J Adv Nurs Stud.

[54] Salah AA, Aljerjawy M, Salama A (2018). Gap between theory and practice in the nursing education: the role of clinical setting. Emergency.

[55] Salifu DA, Gross J, Salifu MA, Ninnoni JP (2019). Experiences and perceptions of the theory-practice gap in nursing in a resource-constrained setting: a qualitative description study. Nurs Open.

[56] Thibeault R, Ann C (2017). Baccalaureate program evaluation, preceptors, and closing the theory-practice gap: is there a connection?. Quality Advancement in Nursing Education-Avancées en formation Infirmière.

[57] Wall P, Andrus P, Morrison P. Bridging the theory practice gap through clinical simulations in a nursing under-graduate degree program in Australia. Int J Learning Teaching Educ Res. 2014;8(1).

[58] Boamah S, Laschinger H (2015). Engaging new nurses: the role of psychological capital and workplace empowerment. J Res Nurs.

[59] Aliakbari F, Parvin N, Heidari M, Haghani F (2015). Learning theories application in nursing education. J Educ Health Promot.

[60] Buck-McFadyen E, MacDonnell J. Contested practice: Political activism in nursing and implications for nursing education. Int J Nurs Educ Scholarsh. 2017;14(1). 10.1515/ijnes-2016-0026 28749781

[61] MacLellan L, Levett-Jones T, Higgins I (2016). The enemy within: power and politics in the transition to nurse practitioner. Nurs Plus Open.

