# Supplementary material for: Nurses’ and midwives’ perspectives on participation in national policy development, review and reforms in Ghana: a qualitative study
Source: BMC Nurs. 2021 Jan 22;20:26. doi: 10.1186/s12912-021-00545-y (PMC7821498; doi:10.1186/s12912-021-00545-y)
Supplement: Supplementary file 1 — Additional file 1. Qualitative interview guide. [file 12912_2021_545_MOESM1_ESM.docx]

**RESEARCH INSTRUMENTS**

**Assessing the contribution of nurses and midwives towards health policy development and review in Ghana (document review/interview guides)**

**QUALITATIVE INTERVIEW GUIDE**

*CODE………………………………..*

**Date of interview………………..**

**Duration………………………….**

**Name of facilitator……………..**

**Name of facility………………….**

**Language used…………………..**

***SECTION A: Biographic Data o***

*Age 18-22 22-26 26-30 30-34 34-38 >38*

*Belief System Christian Moslem Traditional Others*

*Marital status Single Married Co-habitation Divorce*

*Married classification Monogamy Polygamy*

*Years of practice 1-5years 6-10years 11-15years 16-20years >20years*

*Professional group: ………………………………………………………………………….*

*Category of leadership: ……………………………………………………………………...*

**SECTION B- GUIDING QUESTIONS**

1. What are your views on nurses’ and midwives’ participation in national policy development and review?
2. What are the main Health Policy documents for Ghana from 2008 to 2018?
3. How were these policies retrieved?
4. Who were the key contributors and authors?
5. How did Nurses and Midwives contribute to these policies?
   1. Self-initiated by nurses and midwives
   2. “Invitation from policymakers
   3. By-products / end-products of contracts/consultancies
6. What is the trend of Nurses’ and Midwives’ contribution to policy?
   1. Increased or decreased contributions over time
   2. Most/least contribution
   3. Intact verses reviewed policies
7. Who were the Nurses and Midwives playing the Lead roles?
   1. Academic background/level of education
   2. Positions occupied
      1. Operational versus Managerial versus Strategic level
      2. Governmental verses Non-governmental organization (NGO)
8. Which field of nursing practice were the nurses and midwives playing lead roles?
   - 1. Clinical, Education, Research, Education, Policy, Advocacy
9. What was national orientation of the nurses and midwives listed in the report/policy?
   1. Indigenous, Ghanaian based in diaspora, non-Ghanaian national partnering with locals
10. What are some of the policies that you work with as a nurse leader?
    1. Sources
    2. assessment of the policies with direct impact on nursing and midwifery practice
11. What has been your participation in developing or reviewing policy?
    1. Contributions in the past 10 years
    2. Role
    3. Experiences
12. How will you describe the involvement of nurses and midwives in Policy development and review?
    1. Opportunities
    2. Challenges
13. What do you think should be done to ensure nurses and midwives participate in policy development and review?
    1. mentoring the nurses and midwives
    2. Coaching
    3. Other measures
14. Which national health policies do you think need reforms and why?
